# Emotional design pictures: Pleasant but too weak to evoke arousal and attract attention?

**DOI:** 10.3389/fpsyg.2022.966287

**Published:** 2023-01-04

**Authors:** Sina Lenski, Jörg Großschedl

**Affiliations:** ^1^Faculty of Mathematics and Natural Sciences, Institute for Biology Education, University of Cologne, Cologne, North Rhine-Westphalia, Germany; ^2^German Institute for Adult Education, Bonn, North Rhine-Westphalia, Germany

**Keywords:** arousal, attention, electrodermal activity, emotional design, eye-tracking, valence

## Abstract

A new field of research builds on the theoretical assumption that distinct design manipulations, such as human-like features with appealing colors (emotional design), foster multimedia learning by influencing the learners’ affective state (i.e., valence and arousal) and attention. Empirical studies, however, provide inconsistent findings of the affective potential of emotional design, underlining the need for psychophysiological measurements to assess the affective state and attention more objectively. The effects of differently designed stimuli (picture-text combinations with anthropomorphic pictures in bright and saturated colors [emotional design], picture-text combinations with non-anthropomorphic pictures in grayscale [neutral design], and plain text [control design]) on junior high school students’ valence (*N* = 15), arousal (*N* = 18), and attention (*N* = 27) were examined. Valence was determined by students’ judgments on a rating scale; arousal and attention were assessed by psychophysiological parameters (electrodermal activity of students and their dwell time on stimuli, respectively). To allow the examination of valence and arousal as separate dimensions, two independent experiments were conducted. Results of experiment I show that students perceived emotional design stimuli as more pleasant than stimuli in neutral or control design. Besides, an interaction with the content of the stimuli was found. While the positive effect of the emotional design was found for concepts that could be interpreted as potentially positive (e.g., sunlight) or neutral (e.g., consumer), stimuli representing potentially negative concepts (e.g., dead consumer) were not perceived as more pleasant if presented in emotional design. Experiment II shows that emotional design did not induce higher arousal than neutral and control designs and did not attract more attention. Instead, the text within picture-text combinations (emotional and neutral designs) attracted more attention when pictures were presented in neutral than in emotional design. By investigating the emotional state more differentiated and by examining arousal and attention using psychophysiological parameters, the present study helps to understand the heterogeneous findings of previous studies.

## Introduction

In the context of the Covid-19 pandemic, a large amount of educational material (videos, brochures, posters, flyers, and books) has been developed to educate people about the virus and to communicate the code of conduct (e.g., Molina, 2020; Save the Children, 2020; United Nations Children’s Fund (UNICEF) (Producer), 2020). This material often implements design principles that humanize the virus by depicting it with, for example, eyes or mouths (e.g., Centers for Disease Control and Prevention (CDC), 2020). Design principles such as the implementation of human-like features or round shapes and appealing colors lead to a design known as *emotional design*. Emotional design is believed to positively impact learners’ affective state, affecting students’ interest, cognitive load, and learning performance (Um et al., 2012). Meta-analyses, report weak effects of emotional design on students’ affective states but substantial effects on different aspects of learning such as retention, comprehension, and transfers (Brom et al., 2018; Wong and Adesope, 2020). In contrast to the results of the two meta-analyses, the effects reported in individual studies appear to be quite inconsistent. While some studies found the emotional design to change the affective state (e.g., Um et al., 2012; Uzun and Yıldırım, 2018; Shangguan et al., 2020; Bülbül and Abdullah, 2021), other studies found no effects (e.g., Münchow et al., 2017; Münchow and Bannert, 2019; Stárková et al., 2019; Peng et al., 2021). The effect on learning also varies and different factors can cause inconsistent findings. (a) Valence and arousal (activation) were rarely examined as separate dimensions of the affective state, making the multiple results difficult to compare (see, e.g., Heidig et al., 2015 for the use of the PANAVA questionnaire to assess arousal and valence separately). (b) Self-rate questionnaires were the predominant way to gather insight into the affective state, which is error-prone as participants may not be sensitive to their current affective state or changes (Shangguan et al., 2020). Especially arousal seems to be difficult to measure validly by subjective ratings (e.g., Persson et al., 2010). For younger students, Valsiner and Connolly (2003) assume that they are less able to perceive and communicate their affective state. Consequently, several authors call for more objective measures in studying the affective state (e.g., Brom et al., 2016; Bülbül and Abdullah, 2021; Irrazabal and Burin, 2021; Peng et al., 2021). Moreover, previous research (e.g., Moreno, 2006; Moreno and Mayer, 2007; Park et al., 2015) allows the conclusion that the affective state influences attention, and attention, in turn, influences learning. Our study aimed to provide a deeper insight into whether emotional design affects valence, arousal, and attention in junior high school students. The present study results are intended to suggest the practical design of multimedia learning environments in schools.

### Affective state and emotional design

According to Russell’s (2003) *circumplex model*, a spatial metaphor of two orthogonal dimensions can describe individuals’ affective states. The individual’s current position in this two-dimensional space determines the emotion (e.g., anger, happiness, sadness, and relaxation) the individual currently perceives. The first dimension (*valence*) characterizes the value of a stimulus and spans a continuum from the endpoints *pleasant* and *unpleasant*. The second dimension (*arousal*) describes the degree of activation of the central nervous system and spans a continuum between *activated* and *deactivated*. Activating emotions are enjoyment or curiosity (pleasant) and anger or fear (unpleasant). Deactivating emotions include relaxation (pleasant) and sadness (unpleasant, Pekrun et al., 2006). Arousal of the nervous system can be measured by the degree of activation experienced by an individual (by psychophysiological measurements such as electrodermal activity [EDA] or heart rate variability).

Only a few psychophysiological measurements have been used in emotional design research so far. For example, Uzun and Yıldırım (2018) observed participants’ heart rate variability and concluded that emotional design increases arousal. Le et al. (2018) used a similar methodological approach but regarded heart rate variability as an indicator of *mental effort*. They conclude that an emotional design perpetuates mental effort compared to a neutral design. Another methodological approach consists of EDA measurements. EDA measurements have been used in neuromarketing and affective neuroscience for many years but have only recently gained attention in educational research (Braithwaite et al., 2013; Pijeira-Díaz et al., 2016; Caruelle et al., 2019; Posada-Quintero and Chon, 2020). Increased EDA has been measured for stimuli that generate positive emotions, such as preferred products (Ohira and Hirao, 2015), happy music (Krumhansl, 1997), positive facial expressions (Levenson et al., 1990), but for unpleasant stimuli as well (Lang and Bradley, 2007). Findings from these studies suggest that EDA is an indicator of arousal (i.e., the degree of activation) rather than an indicator of valence (Correia et al., 2021).

### Attention and emotional design

Visual *attention* is defined as “the mechanism the nervous system uses to highlight specific locations, objects or features within the visual field” (Bisley, 2011, p. 49). According to the *eye-mind hypothesis* (Just and Carpenter, 1980), it is assumed that the location of eye fixation reflects the place of attention, while fixation duration (so-called *dwell time*) is regarded as an indicator of the amount of attention (Holmqvist et al., 2011). A long-lasting fixation indicates deep information processing or difficulty in processing information (Rayner, 1998), whereas a short-lasting fixation does not. Thus, eye-tracking should give more direct and objective access to cognitive processes (e.g., the amount of cognitive resources devoted to information processing and problem-solving strategies) than self-reports (Alemdag and Cagiltay, 2018). Eye-tracking has been frequently used in multimedia research and provided insight into the processing of information (e.g., van Gog and Scheiter, 2010; Bayram and Bayraktar, 2012; von Reumont and Budke, 2020; Mwambe et al., 2021). A study by Rayner (2009), for example, has shown that the average fixation duration on pictures is longer than fixation duration on text elements. However, Alemdag and Cagiltay (2018) meta-analysis revealed an opposite effect, indicating that learners pay more attention to the text than pictures.

For emotional stimuli, it is assumed that the cognitive system is “likely to be complemented by a perceptual mechanism that is biased to readily detect and process emotional stimuli among other, competing stimuli” (Nummenmaa et al., 2006, p. 257). Eye-tracking research has shown that stimuli emotional stimuli attract more attention compared with neutral stimuli (Nummenmaa et al., 2006). This effect is known as *emotion-related attentional bias* and has been reported in numerous studies (e.g., Harrison et al., 2010). Attention is presumably not caused by valence but by the arousal intensity (e.g., Vogt et al., 2008). However, findings in emotional design research appear to be inconsistent: While Park et al. (2015) found emotional design features to attract attention, Stárková et al. (2019) could not find such an attention-grabbing effect for stimuli adopting emotional design principles compared with non-emotional designs.

## Problem statement

The present study compares the effect of three differently designed stimuli (picture-text combinations with anthropomorphic pictures in bright and saturated colors [emotional design], picture-text combinations with non-anthropomorphic pictures in grayscale [neutral design], and plain text [control design]) on valence (experiment I), arousal, and attention (experiment II) in samples of junior high school students. Whereas valence was determined by students’ judgments on a rating scale, arousal and attention were assessed by psychophysiological parameters (electrodermal activity of students and their dwell time on stimuli, respectively).

The arousal (and attention) and the valence measurements were performed in two separate experiments because the valence self-rating can distort other measurements. Theoretically it is assumed that increased attention that is directed toward the self, will attenuate emotional intensity (May, 1967). It is suspected that attentional resources are allocated to the self at the expense of other activities (e.g., Panayiotou and Vrana, 1998). This means that not only a persons’ ratings but also the resulting awareness of the own emotional state could lead to task irrelevant thoughts and influence a persons’ arousal level.

We assume that the different implementations of emotional design manipulations might be one of the possible causes for the previously mentioned heterogenous findings in the field.

Regarding the previously mentioned heterogenous findings in the field, we assume that one cause could be the different implementation of emotional design manipulations. While in some studies only one design feature was manipulated (e.g., color; Heidig et al., 2015), in others simultaneous manipulations of several features were made (e.g., Um et al., 2012; Uzun and Yıldırım, 2018; Bülbül and Abdullah, 2021). Moreover, only a few previous studies have distinguished between valence and arousal (Chung and Cheon, 2020; Irrazabal and Burin, 2021). As these findings are mainly based on self-rating, physiological measurements could provide more valid results. Thus, we based our hypotheses on theoretical assumptions and findings using EDA and eye-tracking measurements.

*Hypothesis 1 (H1):* Stimuli in emotional design will be perceived as more pleasant (measured by self-rating) than those in neutral or control designs.

*Hypothesis 2 (H2):* Stimuli in emotional design lead to a higher state of arousal (measured by EDA) than those in neutral or control designs.

*Hypothesis 3 (H3):* Pictures in emotional design will attract more attention (measured by dwell time) than those in a neutral design.

*Exploratory Goal* (EG): Textual elements in picture-text combinations attract more attention than pictorial elements. An explorative approach aimed to investigate whether the pictorial element’s emotional design (vs. neutral design) affects this preference.

## Experiment I

### Method

#### Sample

A total of 23 German junior high school students from grades 8 and 9 were recruited. Eight students had to be excluded as they did not complete the questionnaire. The remaining sample consists of *N* = 15 students (53% female; *M*_age_ = 13.67 years, *SD* = 0.62).

#### Stimulus material, design, and procedure

**Figure 1:**
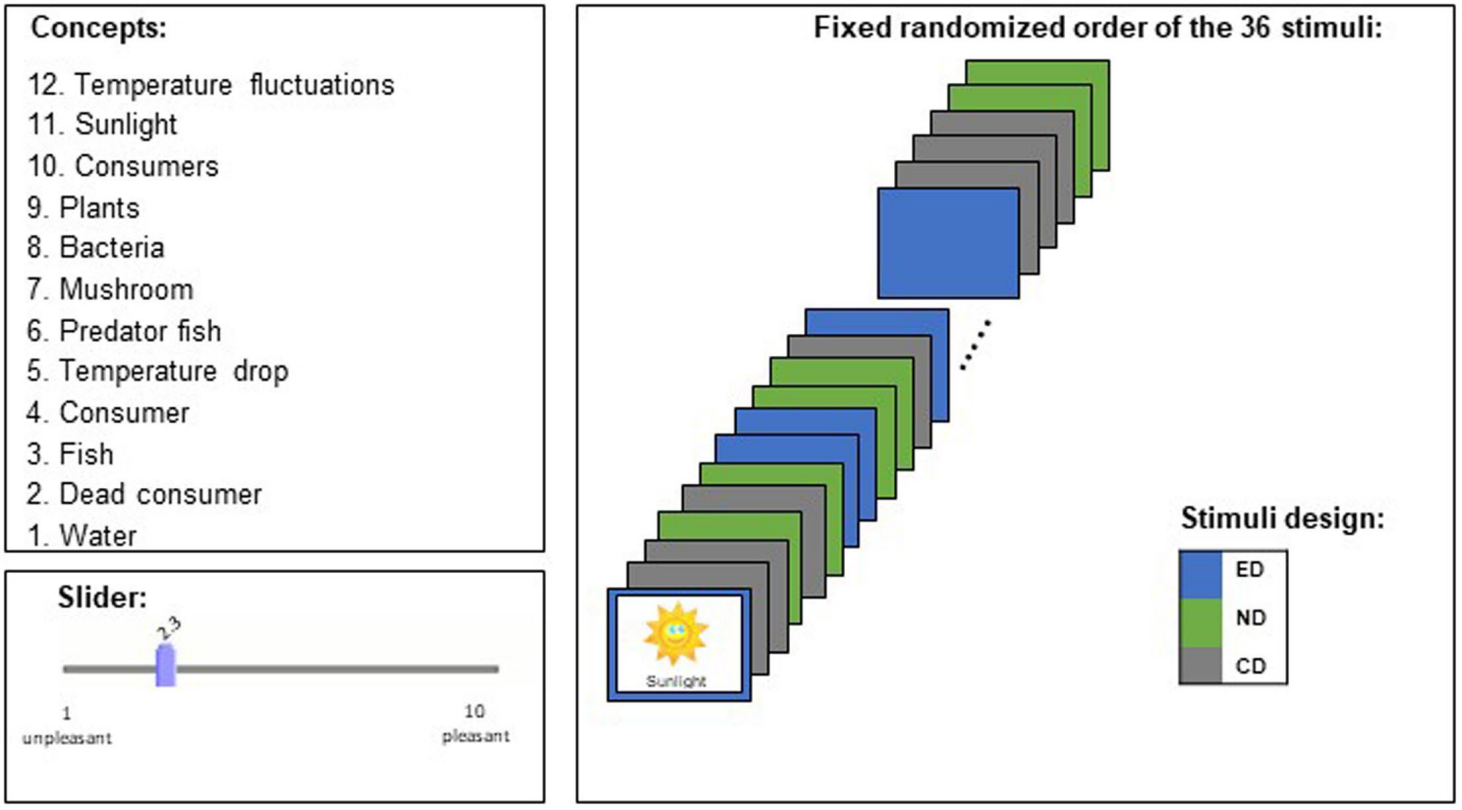
Research design and procedure for experiment I. All participants rated the valence of 12 concepts in three different designs (emotional design [ED], neutral design [ND], and control design [CD]; 36 stimuli). The stimuli varied in a randomized order.

We selected twelve concepts (e.g., *water*, *fish*, and *consumer*) of a biological topic (Lake Ecosystem) and applied three design principles to represent them. The application of the design principles resulted in 12 × 3 stimuli (nota bene: we refer to a concept that is presented in a particular design as a stimulus), of which 12 represented an emotional design, 12 a neutral design, and 12 the control design (stimulus material is available as Supplementary material S1) and information on the procedure is visualized in Figure 1). Emotional stimuli are picture-text combinations with anthropomorphic pictures in bright and saturated colors. Previous studies showed that the manipulation of color and the use of anthropomorphisms in combination showed greater effects on emotions than the use of color or the use of anthropomorphisms alone (e.g., Um et al., 2012; Uzun and Yıldırım, 2018). Accordingly, we applied the design principles defined by Um et al., 2012 who coined the term “Emotional Design.” Neutral stimuli are picture-text combinations with non-anthropomorphic pictures in grayscale, and control stimuli are plain text without a pictorial representation. An online survey was carried out on the *SoSci Survey* platform and shared with the participants on www.soscisurvey.com. The survey started with instructional information, followed by demographic data (age, gender, and grade level). Then all 36 stimuli were presented in a randomized order with one stimulus per page. Below each stimulus, a slider scale was displayed to gain information about perceived valence. The participants were asked to look at the stimuli and evaluate them intuitively, using the slide scale. They were informed that they had to select to proceed and that the survey should be worked on alone in silence. The program did not allow to continue without answering, and the survey took an average time of *M* = 3.39 min to complete (*SD* = 0.59 min, range 2.42–4.42 min).

#### Instrument

To examine the effect of different design principles (emotional, neutral, and control design) on valence, a ten-point slider scale from *unpleasant* (rating = 1) to *pleasant* (rating = 10) was placed below each stimulus. A similar rating scale was used by Yao et al. (2017).

### Results

According to our first hypothesis (H1), stimuli in emotional design are more pleasant than stimuli in neutral design or control designs. A 12 × 3 ANOVA (rANOVA) with the two within factors *concept* (temperature fluctuations, sunlight, consumers, plants, bacteria, mushroom, predator fish, temperature drop, consumer, fish, dead consumer, water) and *design* (emotional, neutral, control) was conducted to explore the effect of different designs on valence ratings, taking the concepts of the stimuli into account (see Table 1 for descriptive data). The rANOVA with Greenhouse–Geisser adjustment revealed a significant main effect of *design* on participants’ valence ratings, *F*(1.17, 16.32) = 69.03, *p* < 0.001, *η_p_*^2^ = 0.831 (strong effect with *f* = 2.22). Bonferroni-corrected pairwise comparisons showed that emotional design stimuli (*M* = 6.78, *SD* = 0.87) appear more pleasant (i.e., higher valence ratings) than neutral stimuli (*M* = 4.48, *SD* = 0.47) and control stimuli (*M* = 4.73, *SD* = 0.66), thereby supporting H1.

**Table 1:** Mean valence ratings and standard deviations for design (*n* = 15).

	ED		ND		CD	
Variable	*M*	*SD*	*M*	*SD*	*M*	*SD*
Valence	6.78	0.87	4.48	0.47	4.73	0.66

ED = emotional design; ND = neutral design; CD = control design.

**Figure 2:**
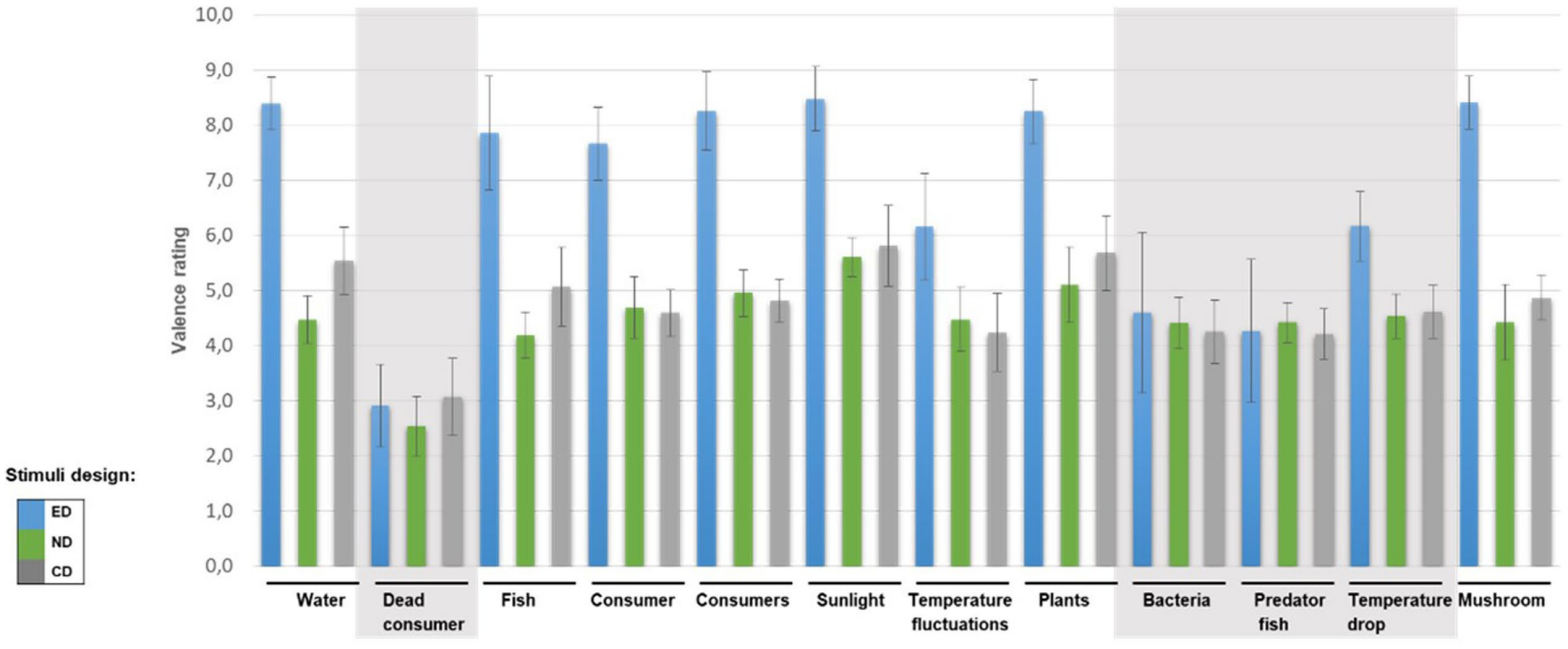
Mean valence rating and standard deviations for different concepts in emotional design (ED), neutral design (ND), and control design (CD). The higher the valence ratings, the more pleasant the concept is perceived. Concepts that differ significantly in valence ratings from the other concepts are highlighted in grey. Highlighting is based on *post-hoc* analyses which can be viewed in the Supplementary material S2.

Also the effect of *concepts* is large (*f* = 1.56) and significant, *F*(3.08, 43.09) = 33.95, *p* < 0.001, *η_p_*^2^ = 0.708, which indicates that the concept of a stimulus also influences students’ valence rating. Valence ratings for some concepts (e.g., dead consumer) are significantly lower than for other concepts (e.g., sunlight; see Figure 2). In addition, the rANOVA revealed a significant interaction effect between design and concept, *F*(5.30, 74.15) = 12.39, *p* < 0.001, *η_p_*^2^ = 0.470 (strong effect with *f* = 0.942). A closer look at the data showed that the design affects valence ratings for some concepts (e.g., sunlight) but not for others (e.g., dead consumer; see Figure 2, grey highlights). It is worth mentioning the high scatter of the data for the concepts of bacteria and predatory fish in the emotional design. Descriptive data and post-hoc analyses for the concepts are provided in the supplementary material (Supplementary material S2).

To determine the minimum effect size that could reliably yield a statistically significant result based on the remaining sample size, we conducted a sensitivity analysis using G*Power. A two-way within-subjects ANOVA (12 × 3) with 15 participants across three conditions would be sensitive to effects of *f* = 0.55 with 95% power (alpha = 0.05).

### Interim discussion

The valence measurement aimed at investigating whether stimuli in emotional design are perceived as more pleasant than non-emotional design stimuli. As expected, the emotional design increased the perceived pleasantness (higher valence ratings) compared to the neutral and control designs (*cf*. H1). Interestingly, this is true only for concepts representing positive or neutral content (e.g., sunlight, plants, and fish). Valence ratings did not differ between emotional, neutral, and control designs in the case of concepts representing potentially negative content (e.g., dead consumer, bacteria, and predator fish).

One reason could be that the emotional design increases perceived pleasantness through positive facial expressions (e.g., warm colors and round face-like shapes). In contrast, negative facial expressions (e.g., saturated red hues and angry eyes) might not increase the perceived pleasantness of a stimulus to the same extent however did not lead to more significant lower ratings compared to other design either. A similar result has been reported by Navratil et al. (2018), who observed that students rated the pleasantness of intended-positive emotional design higher than the pleasantness of intended-negative emotional design. This effect, however, was only observed when the students were exposed to both conditions (within-subjects design). If the students were assigned to one condition only (between-subjects design), the intended-positive and intended-negative emotional designs were perceived equally positively. Furthermore, Navratil et al. (2018) presume that learners prefer design manipulations that are congruent with the design, meaning that a concept representing negative content should be depicted in a negative emotional design. A positive emotional design representing negative content could be perceived as inappropriate and affect the valence negatively. It is also possible that the facial expressions are perceived or interpreted differently.

Another reason why the effect of the emotional design is lower for potentially negative stimuli could be that for negative stimuli associations or previous experiences could have a greater impact here. Perhaps the concept of bacteria is rated negatively by some students because they think of pathogens, while others associate them with the intestinal flora which is important for health. This is supported by the fact that for stimuli with negative content the spread of the valence ratings was high and therefore data less homogeneous.

The results of our study suggest that the valence of the content can influence the effect of emotional design. So far, there are no studies that examine the interaction between content and design. For future research, we recommend the systematic variation of positive and negative emotional design for positive and negative content. This differentiation could provide further explanations for the previously heterogeneous findings and lead to the creation of more precise design recommendations.

## Experiment II

### Method

#### Sample

A total of 30 German junior high school students from grades 8 and 9 (58% female; age: *M* = 14.31, *SD* = 0.54) were recruited for experiment II. They received small gifts as an expense allowance (e.g., pens or candy bars). Three students refused the EDA measurements, another three students produced incomplete data, and six students were non-responders (no usable SCR, see below). This results in an adequate sample size of *n* = 18 for H2 testing. Three students had to be excluded for eye-tracking measurements because of measurement errors or disturbances during the experiment, which resulted in a sample size of *n* = 27 students for H3 testing.

#### Stimulus material, design, and procedure

**Figure 3:**
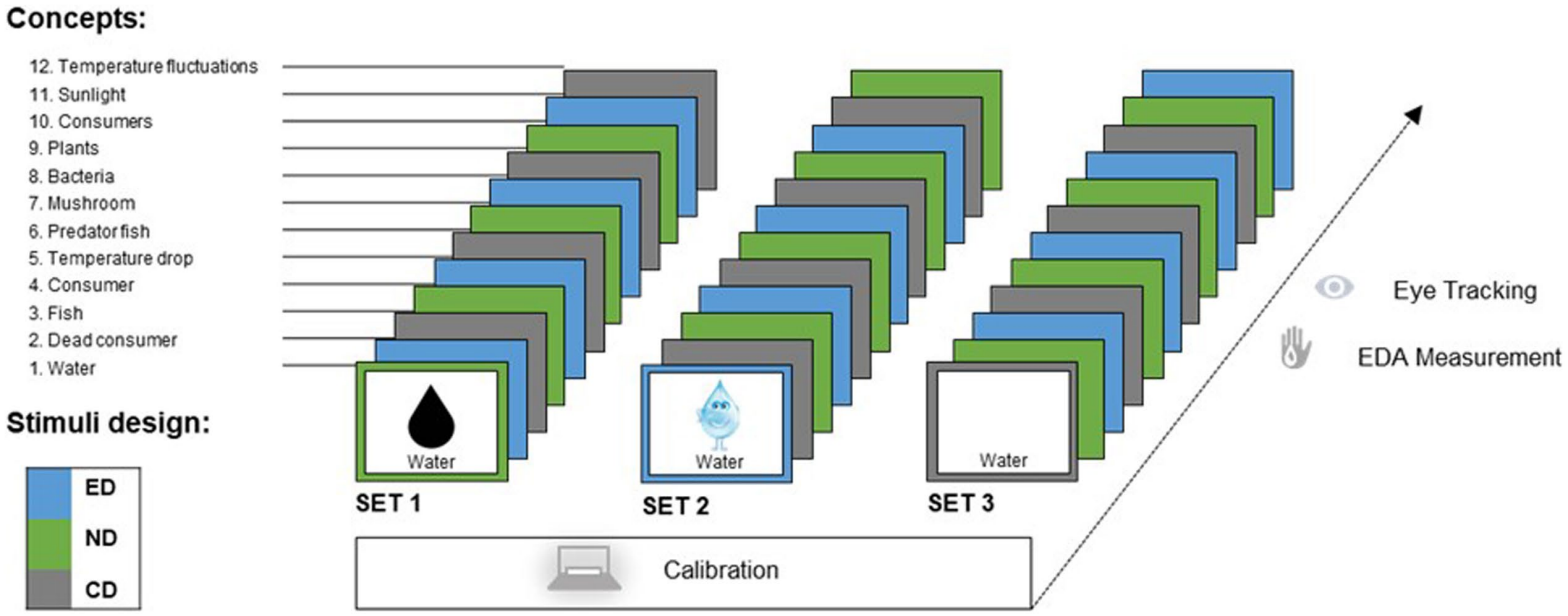
Research design and procedure. Students were assigned to one of three sets of stimuli. The 12 concepts (left) were displayed, with their design varying from one stimulus to the next (emotional design [ED], neutral design [ND], and control design [CD]).

In experiment II, students were randomly assigned to one of three sets of 12 stimuli (nota bene: we refer to a concept [e.g. *water*; see Figure 3] that is presented in a particular design [emotional, neutral, or control design] as a stimulus). In each set of stimuli, the order of the concepts (water, dead consumer, and fish, etc.) was identical. Still, the design changed in a fixed order so that each concept was presented for 8 s in each set in a different design (e.g., the first concept *water* in set 1 in neutral, in set 2 in emotional, and in set 3 in control design; see Figure 3). A neutral stimulus was followed by an emotional stimulus, an emotional stimulus followed by a control stimulus, and a control stimulus followed by a neutral stimulus. Before the experiment started, the students were given a brief overview of the procedure, and the EDA sensor was attached to the wrist of the student’s non-dominant arm. The eye-tracking system was then calibrated for each student using 5-point-calibration (viewing distance was approximately 15.7–19.7 inches). Immediately after the calibration, the instructor started the experiment. The duration of the whole session was about 15 min per student. We chose this comparatively complex research design to avoid order, minimize fatigue effects, and reduce the influence of the concept represented in evaluating the impact of the design. The stimulus material is available as supplementary material (Supplementary material S1).

#### Psychophysiological measurements and instruments

Several techniques (e.g., EDA measurements) are currently available which use psychophysiological parameters to measure arousal (Dzedzickis et al., 2020). EDA reveals implicit emotional responses that may occur without conscious awareness and is believed to provide an objective assessment of arousal (Braithwaite et al., 2013). EDA is considered the only psychophysiological parameter that is sensitive to changes in emotional state and is not influenced by the activity of the parasympathetic system (Braithwaite et al., 2013). When an emotionally arousing stimulus is experienced, the skin moisture level changes due to an increase in the activity of eccrine sweat glands. The resulting amplification in electrical properties of the skin, the EDA, can reveal changes in the sympathetic nervous system.

EDA consists of two essential signals: the slowly changing tonic signal, skin conductance level (SCL), and the rapid changing phasic signal, skin conductance response (SCR). The SCL is a background signal, as only slight changes occur over minutes (Dzedzickis et al., 2020). SCRs arise in response to a single stimulus (event-related, ER-SCRs) but can also occur spontaneously (nonspecific skin conductance responses [NS-SCRs]; Boucsein, 2012). The onset of the ER-SCR typically occurs between 1 and 4 s after stimulus presentation (Dawson et al., 2017), which means that “any SCR that begins between one and three (or four) seconds following stimulus onset is considered to be elicited by the stimulus” (Boucsein, 2012, p. 207). The parameters *ER-SCR amplitude* and *ER-SCR count* are used as indicators for arousal (Braithwaite et al., 2013). An *EdaMove 4* sensor (Movisens GmbH, Karlsruhe, Germany) was used with two self-adhesive Ag/AgCl electrodes. EDA was recorded with a sampling rate of 32 Hz by a constant voltage system (0.5 V).

**Figure 4:**
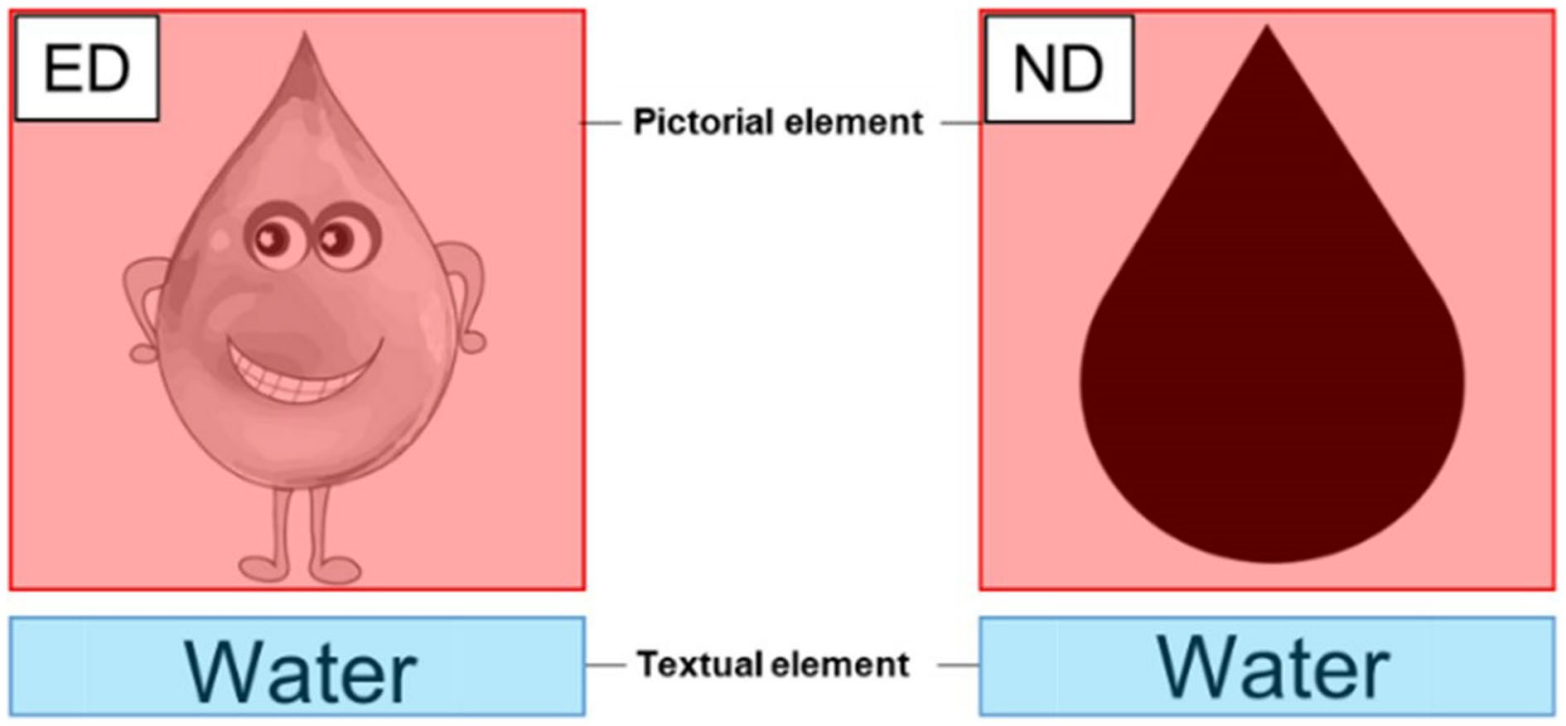
Sample presentation of areas of interest (AOIs) for the stimulus *water* (translated from German) in the emotional design (ED) and the neutral design (ND). Red: AOI_pictorial_; blue: AOI_textual_.

Eye-tracking measurements were carried out to examine the influence of different design conditions on attention, with *dwell time* as an indicator (see Stárková et al., 2019 for a similar approach). Here, dwell time is understood as the sum of durations of all fixations and saccades that hit an *Area of Interest* (AOI). An AOI is a delimited two-dimensional space on a stimulus for which metrics should be extracted (see Figure 4). To account for different sized AOIs, all dwell time values reported are normalized (dwell time in milliseconds [ms] divided by AOI coverage). This way, it is a reliable measurement, as it adjusts the time spent to process an AOI to its relative surface in the screen (Ceravolo et al., 2019). The eye-tracking system *SMI RED250 mobile* (SensoMotoric Instruments GmbH, Teltow, Germany) was used on a 17-inch screen to measure the students’ dwell time on different AOIs. As it is common in eye-tracking research, only the right eye position was analyzed (Lykins et al., 2006). According to the manufacturer, data were recorded at 60 Hz with an accuracy of 0.4°, and head movements were not restrained. *IViewХ* was used for data collection, and stimuli were presented by *Experiment Center 3.0* (SensoMotoric Instruments GmbH, Teltow, Germany). Fixations were detected using the default dispersion-based algorithm (100 pixels and a minimum duration of 80 ms).

#### Signal processing

**Figure 5:**
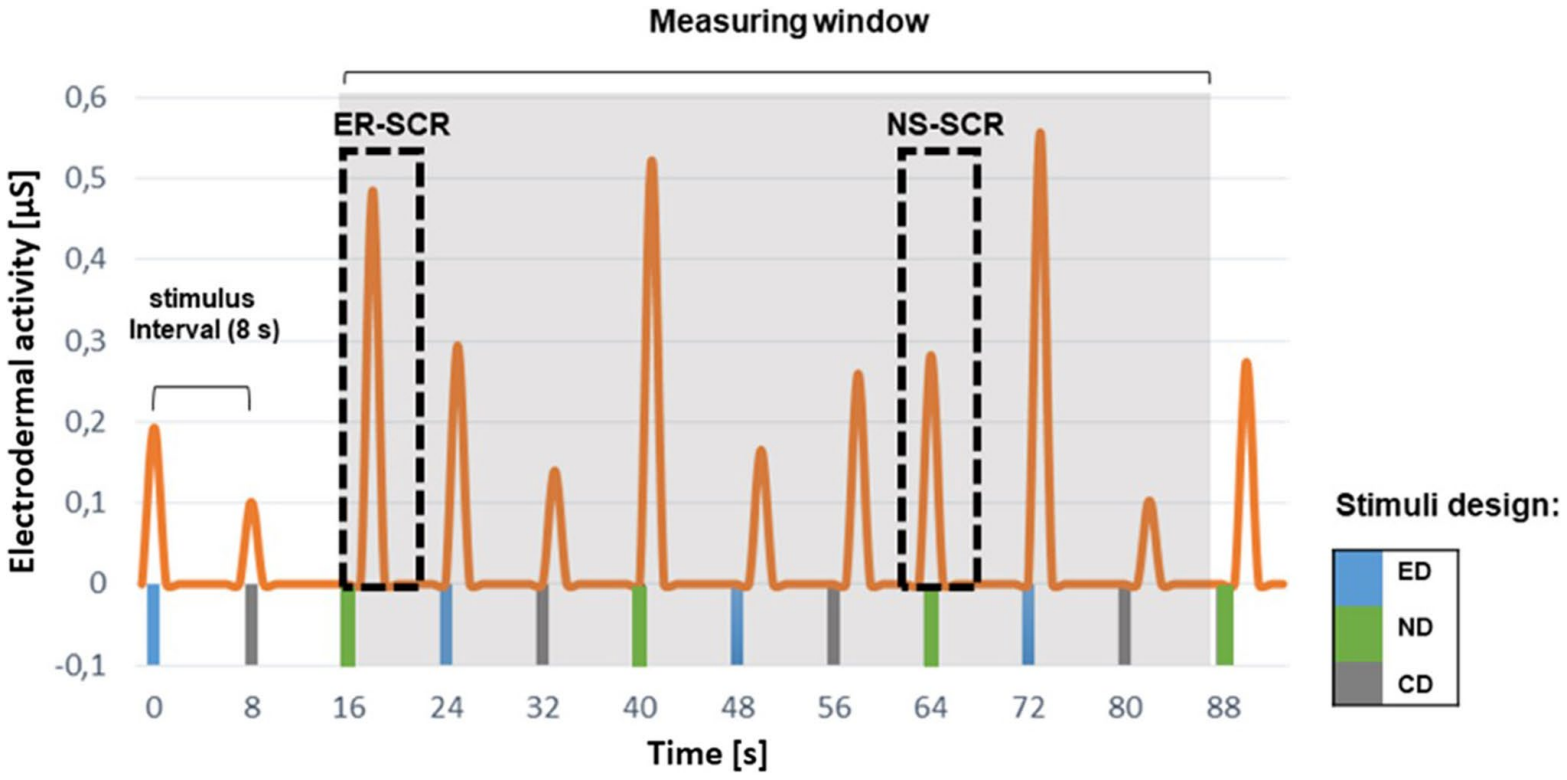
Exemplary skin conductance response (SCR) signals for set 2. The measurement window comprises nine stimuli (3× emotional [ED], 3× neutral [ND], and 3× control design [CD]). Each stimulus is being viewed for 8 s. A signal which occurred within the chosen response window (1–3 s) is rated as an event-related SCR signal (ER-SCR). In contrast, a signal that occurred outside the response window is considered a non-specific SCR signal (NS-SCR).

EDA data (*SCR amplitude* and *SCR count*) were imported to IBM SPSS Statistics (version 24.0) using *SensorManager*, viewed and preprocessed using *UnisensViewer,* and analyzed using *DataAnalyzer* (*Movisens* GmbH, Karlsruhe, Germany). To test H2, each student’s SCR amplitude and SCR count related to a specific design (emotional, neutral, or control design) were averaged. The first, second, and twelfth stimuli presented to the students were excluded from the analyses because the beginning and the end of the EDA measurements needed to be marked by tapping the device, which could cause SCRs not related to the treatment (see Figure 5). Hence, *SCR amplitude* and *SCR count data* for nine stimuli and each student (3 x emotional, 3 x neutral, and 3 x control design) remained for analyses (e.g., for neutral design in set 1: consumer, mushroom, consumers).

To extract SCR data, raw data were exported to *UnisensViewer*, automatically detecting SCR. Default settings by *Movisens* firmware to see SCRs (minimal rise time of 0.05 μS/s, minimal amplitude of SCR of 0.1 μS, and a maximal rise time of SCR of 0.9 s) were used based on Dawson et al. (2017) and Boucsein (2012). Output intervals were set, and datasets were checked for artifacts that led to the exclusion of the respective students. ER-SCRs were classified and considered in the analyses when they occurred within a response window of 1 to 3 s following stimulus onset (Braithwaite et al., 2013; Dawson et al., 2017). This narrow response window was applied to reduce the possible contamination of measurements by NS-SCR signals. The parameters (*ER-SCR amplitude* and *ER-SCR count*) in the response window were chosen for hypothesis testing (see Figure 3) as Dawson et al. (2017) recommended.

Eye-tracking data were viewed and analyzed using *BeGaze* (*SensoMotoric Instruments GmbH*, Teltow, Germany) for all 12 stimuli. AOIs for the stimuli consisting of pictorial and textual elements (emotional design and neutral design) were pre-defined. Pictures were defined as AOI_pictorial_, while all textual elements were defined as AOI_textual_ (see Figure 4). Overlapping of AOIs was avoided, and margins were left between different AOIs (Holmqvist et al., 2011).

All students’ eye-tracking and EDA data (from the three sets) were combined into one data set. Mean values of the different parameters (SCR count, SCR amplitude, and dwell time) were calculated for each design (emotional design, neutral design, and control design). This approach is considered valid, as the set allocation was randomized, and all test conditions were kept constant.

To test H3, attention (measured by *dwell time*) on pictorial and textual elements was averaged for each student resulting in four dependent variables (dwell time ED-AOI_pictorial_, dwell time ED-AOI_textual_, dwell time ND-AOI_pictorial_, dwell time ND-AOI_textual_). Dwell time ED-AOI_pictorial_ and dwell time ND-AOI_pictorial_ provide information about a student’s attention to pictorial elements in emotional design (ED) and neutral design (ND), respectively. Dwell time ED-AOI_textual_ and dwell time ND-AOI_textual_ describe a student’s attention spent on textual elements presented in combination with a pictorial element in emotional or neutral design (see Figure 4).

### Results

We used a one-factorial design to examine H2 and H3. While for H2 the three different designs (emotional design, neutral design, control design) were examined, for H3, only the designs consisting of picture-text combinations were focused on (emotional design, neutral design). Because the control design consisted only of text elements, it was excluded. As the data did not meet assumptions for parametric analyses, distribution-free nonparametric analyses were performed.

#### Arousal

EDA measurements investigate whether different design principles affect arousal. According to H2, stimuli in the emotional design are expected to evoke emotions, leading to a higher state of arousal compared with concepts in the neutral and the control designs. For EDA data in the response window (see Figure 5), Friedman’s test with the within-factor *design* (emotional, neutral, and control) revealed no significant difference for *SCR amplitude* (χ^2^[2] = 0.03, *p* = 0.220) and *SCR count* (χ^2^[2] = 3.06, *p* = 0.217) among the different design conditions (lack of support for H2). Descriptive data are shown in Table 2.

**Table 2:** Mean ER-SCR amplitude, count, and standard deviations for design (*n* = 18).

	ED		ND		CD	
Variable	*M*	*SD*	*M*	*SD*	*M*	*SD*
ER-SCR amplitude (μS)	0.16	0.14	0.11	0.14	0.23	0.21
ER-SCR count (*n*)	0.35	0.27	0.26	0.27	0.44	0.38

ED = emotional design; ND = neutral design; CD = control design; ER-SCR = event-related skin conductance response.

A sensitivity analysis revealed that a one-way within-subjects ANOVA with 18 participants across three conditions would be sensitive to effects of *f* = 0.25 with 95% power (alpha = 0.05) with a correlation among the repeated measured of *r* = 0.8. As we applied Friedman’s test instead of a one-way within-subjects ANOVA we need to correct for the use of a nonparametric test manually. The most common index for comparing nonparametric tests to parametric tests is asymptotic relative efficiency (Prajapati et al., 2010). Applying adjustments, the Friedman’s test, the power would be 0.72 instead of 0.95 to detect an effect of *f* = 0.25.

#### Attention

Regarding H3, it is presumed that pictorial elements in the emotional design attract more attention than pictorial elements in a neutral design. A Wilcoxon signed-rank test revealed that students’ dwell time on pictorial elements in the emotional design (*M* = 21862.92 ms/coverage, *SD* = 966.41) was not greater than that on pictorial elements in the neutral design (*M* = 22054.07 ms/coverage, *SD* = 997.95), *Z* = −1.92, *p* = 0.848 (lack of support for H3). The Wilcoxon signed-rank test with 27 participants would be sensitive to effects of *d* = 0.53 with 95% power (alpha = 0.05).

Referring to the exploratory goal (EG) of our study, results show textual elements attract more attention (*M* = 31770.42 ms/coverage, *SD* = 3307.93) compared to pictorial elements (*M* = 21958.50 ms/coverage, *SD* = 859.95), *Z* = −2.31, *p* = 0.042 (strong effect with *d* = 0.992). Additionally, textual elements which were co-represented with neutral design pictures (*M* = 36091.51 ms/coverage, *SD* = 4025.91) attracted significantly more *attention* than those that were presented together with emotional design pictures (*M* = 27449.33 ms/coverage, *SD* = 2909.25), *Z* = −3.22, *p* = 0.002 (strong effect with *d* = 1.579). Bonferroni-Holm correction was applied. Descriptive data are shown in Table 3.

**Table 3:** Mean dwell times and standard deviations for design (*n* = 27).

Design	ED		ND	
	*M*	*SD*	*M*	*SD*
Dwell time [ms/coverage] AOI_pictorial_	21862.92	966.41	22054.07	997.95
Dwell time [ms/coverage] AOI_textual_	27449.33	2909.25	36091.51	4025.91

ED = emotional design; ND = neutral design; AOI_pictorial_ = area of interest represents attention on pictorial elements; AOI_textual_ = area of interest represents attention on textual elements.

### Interim discussion

This experiment aimed at providing additional objective insights into whether emotional design influences *arousal* and *attention*.

#### Arousal

The aim of the EDA measurements was to examine the effect of different designs on arousal using psychophysiological responses of the skin. Stimuli in the emotional design were expected to cause higher arousal compared to other design manipulations. However, the obtained results of this study did not support the hypothesis (lack of support for H2). Our findings are in line with several other studies (Park et al., 2015; Münchow and Bannert, 2019) were also unable to find effects of emotional design on the affective state. Stárková et al. (2019) assumed the affective influence of emotional design to be small in general and difficult to detect (supported by the meta-analysis of Wong and Adesope, 2020) and that the affective effect might be influenced by other variables that have not been identified yet.

The descriptive EDA findings yielded the following results: emotional design (0.16 μS), neutral design (0.11 μS), and the control design (0.23 μS). In a study by Choi et al. (2012) examining virtual agents with different facial expressions, maximum values (for 6-s trends) of around 0.25 μS were obtained for the joy and anger conditions, whereas for a neutrally designed agent the value was around 0.15 μS. Khalfa et al. (2002) examined video clips assigned to different valences and showed that fearful and happy music resulted in higher SCR values (values around 0.25 μS) compared to sad and peaceful music (values around 0.15 μS). In contradiction to this, in our study not only SCR values were not higher for the emotional design but even the results of the control group were the highest. One reason could be that words alone (as in the control design) may elicit strong emotional reactions as the word leaves room for a stronger emotional association. The word “sunlight” for example could trigger a positive association with the last summer vacation and thus cause higher arousal, possibly even higher than the anthropomorphized sun depicted in the emotional design. The emotional design could thus create less space for associations and thus also lead to less arousal.

In addition to the possibility that the control design could have been more arousal-inducing, it could be suspected that the emotional design manipulation was too weak. An aspect that could influence arousal is whether the emotional design is presented statically or animated and what degree of humanization is used. Previous studies in which emotional design increased the arousal (measured *via* heart rate variability) applied either animated stimuli with narration (Le et al., 2018) or static stimuli with sound effects (Uzun and Yıldırım, 2018; Bülbül and Abdullah, 2021). They could be seen as more complex emotional designs compared to the basic emotional design manipulation used in this present study and in other studies (e.g., Um et al., 2012; Münchow et al., 2017). At present, it is not entirely clear which design criteria must be fulfilled by an emotional design to evoke emotions. Characteristics that may vary in pictorial emotional design include colors, sounds, shapes, and humanization of objects. Thus, studies summarized under the term emotional design incorporate investigations in which only one characteristic was manipulated (color, Heidig et al., 2015) or subtle emotional interface design was applied (Peng et al., 2021) but also those where many characteristics were varied (Uzun and Yıldırım, 2018; Bülbül and Abdullah, 2021).

In our experiment, the design manipulations recommended by Um et al., 2012 were applied on static pictures. One reason why these manipulations had no influence on arousal could be that this kind of design was nothing new for the participants. It can be assumed that children and adolescents are already familiar with the design manipulations used in emotional design through modern entertainment like films and (computer) games. A high level of familiarity could mean that the emotional design must be more intensively designed (e.g., using various design manipulations and animations) in order to induce arousal and attract attention (the feasibility of such elaborate designs is discussed in the outlook section). For adults, this familiarity could be lower and thus the novelty effect could play a greater role here. Emotional design manipulations have so far mainly been studied in students. A possible novelty effect should be excluded in future studies, as its influence may not be sustainable. Therefore, age and familiarity with emotional design should be considered as possible influencing factors in future studies.

#### Attention

The aim of the eye-tracking measurements was the examination whether stimuli in emotional design attract more attention compared to stimuli in neutral design. Contrarily to the expectation, results show no differences in dwell time between pictures in the emotional and the neutral design (lack of support of H3). These results are conflicting with the *emotion-related attentional bias* but are in line with findings by Stárková et al. (2019), who could not find a significant difference for the total dwell time comparing emotional design and neutral design pictures. However, they observed that emotional design pictures attracted significantly more attention during initial observation (first 2 s of viewing). Park et al. (2015) found longer overall fixation durations for emotional design pictures (expressive anthropomorphisms) compared with pictures in a neutral design. There are various possible reasons for these conflicting results. Differences in the implementation of emotional design could cause differences in arousal intensity, as already discussed for the EDA measurements. Furthermore, the results suggest that textual elements attracted more attention than pictorial elements in picture-text combinations, corresponding with findings by Stárková et al. (2019) and the meta-study by Alemdag and Cagiltay (2018). Interestingly, the explorative examination revealed that textual elements attracted more attention when the co-represented pictures were shown in a neutral design compared with pictures in an emotional design (EG). One possible explanation could be that the abstractness of the neutral design pictures may cause difficulty in recognizing the concept visualized. To compensate this lack of information, an intense study of the textual element is necessary. Emotional design pictures include colors and more details which could facilitate the recognition of the depicted concept. Supporting this, Mason et al. (2013) claimed that “more detailed iconic diagrams […] are less abstract and closer to the referent they depict … [and] are potentially easier to interpret” (p. 358). Inline, Lin et al. (2017) found that “readers focused more on the text when learning with simplified illustrations” (p. 578). Another explanation could be based on the limited capacity assumption. Colors and humanizations presented in the emotional design pictures may be more cognitively demanding regarding picture-text integration compared to the neutral design. Therefore, the extraneous cognitive load could be higher for emotional design stimuli reducing the capacity available for text processing [see, e.g., (Sweller (2005) for more information on cognitive load]. Similarly, Koedinger et al. (2008) found that abstract representations put fewer demands on working memory compared to detailed representations. Cognitive resources available for a task are decisive for learning success (Sweller, 2005). Therefore, future studies should investigate the effect of different picture-text co-representations on different types of cognitive load.

## Summary

In this study, static pictures applying a basic emotional design as developed and evaluated by Um et al. (2012) were created. From the findings it can be concluded that the static emotional design influences the affective state *via* the valence dimension. However, this does not seem to apply to negative content and correspondingly designed stimuli (use of negative facial expressions). In agreement with the presumption that the emotional design should be coherent with the content (Navratil et al., 2018), future studies should address a potential content-and coherence-dependent effect of emotional design manipulations. No differences could be found for the arousal dimension compared to non-emotional designs. Although emotional design stimuli were perceived as more pleasant, they did not attract more attention compared to neutral stimuli in the present study. This is supported by the presumption of Vogt et al. (2008) that attention is not driven by valence but by arousal intensity.

Emotional design represents an approach with great potential within multimedia learning. The present study represents an important step towards more objective measurements and sets new impulses in the field of emotional design.

## Limitations

Strength and limitation at the same time is the fact that this study was not carried out as part of a learning setting. Disruptive influences generated by a learning process could be reduced but comparing the results of the present study to those of previous studies is only possible to a limited extent. Since a learning process is more complex than the mere viewing of different stimuli, the emotional design could have a beneficial, for example, an attention-directing effect in learning settings, i.e., a *cueing effect* (van Gog and Scheiter, 2010) which was not possible to examine in the present study. The design chosen in experiment II should reduce order effects focusing boredom and fatigue. For future studies an additional randomization (e.g., ABBA reverse counterbalancing or block randomization) would be recommended to achieve a complete balancing. Because this study used within-subjects designs., it is possible that carry-over effects or other effects may have occurred because of participants being able to compare the different designs. This should be considered for future studies although especially for the EDA measures, we assume this to be less relevant and would not recommend between-designs as these values are more difficult to compare interpersonally (see Dawson et al. [2017] for more information). EDA measurements are “a sensitive index of emotional processing” (Braithwaite et al., 2013, p. 3) but aspects of analysis could reduce validity. To assign arousal to the individual stimuli (identify ER-SCR), a too strict sorting out of supposedly unspecific signals (NS-SCRs) can harbor the risk of missing ER-SCRs. This misclassification of NS-SCR signals could lead to a reduction of sensitivity (Braithwaite et al., 2013). To avoid those errors in future studies, data analysis using software particularly suited to determining ER-SCRs (e.g., *BIOPAC MP36R* or *Acq Knowledge* Software) is recommended. To enable a better assignment of the ER-SCRs to the individual stimuli, intermissions between the individual stimuli (blank screens) should be applied. For H2 and H3 nonparametric measures had to be applied. Sensitivity analyses however suggest that the analyses were sensitive enough to detect effect sizes reported in previous studies (*f* = 0.40 for emotional state variables [e.g., Um et al., 2012; Plass et al., 2014; Uzun and Yıldırım, 2018]; *d* = 0.55 for attention [Park et al., 2015]). However, it must be noted that this study was designed to detect medium to large effects. As the effects of the static emotional design on attention and arousal might be small, we recommend larger sample sizes. The high drop-out rate (non-responders and measuring faults) should be considered in sample acquisition.

In this light, the findings of the present study and related interpretations should be treated context-related and further research is needed in this area.

## Outlook

The emotional design of educational material is particularly interesting for school learning as its effect on affective state and learning is supposed to be high especially in younger age groups (Schneider et al., 2019; Wong and Adesope, 2020). Most studies in the field have so far been carried out on college students (Brom et al., 2018). Therefore, it is unclear whether previous results are applicable to younger students. Furthermore, Schneider et al. (2019) suspect that there is “an optimum range of arousal, which is not too low (no activation) and not too high (avoidance behavior), but instead activates learners” (p. 73). An emotional design apparently needs a certain intensity to induce arousal, but should not be too strong, otherwise the stimulus could have a distracting effect (see seductive detail effect; Park et al., 2011). Supporting this, Bülbül and Abdullah (2021) achieved convincing effects on emotions and educational variables such as transfer, interest, and intrinsic motivation for a complex, intense emotional design animation. Static pictures based on the emotional design criteria of Um et al. (2012), however lead to conflicting results regarding their influence on emotions and educational variables and is suspected to be not intense enough to induce emotions and attract attention (e.g., Stárková et al., 2019; Bülbül and Abdullah, 2021).

Moreover, although all material that have been examined so far in emotional design research can be classified as multimedia learning material, a more detailed classification is needed. Weidenmann (2002) proposed a classification into three different categories: *multimediality*, *multicodality*, and *multimodality*. Multimedial is characterized by the integrated use of technical devices, such as a computer and DVD player, while multicodal refers to the use of various symbolic systems (e.g., combinations of picture and text). Meanwhile, a single picture or text is classified as monocodal. Audio-visual learning software consisting of pictures and sounds would be categorized as multimodal because various sensory modalities are addressed (sight and hearing; for more information see Weidenmann, 2002). According to this categorization, it is not only the complexity/intensity of the emotional design that must be considered but also the way in which it is presented. If this classification is applied to previous studies on emotional design, mainly monomedial (computer-based) and primarily multicodal settings (Münchow et al., 2017; Münchow and Bannert, 2019; Stárková et al., 2019; Shangguan et al., 2020) or multicodal*-*multimodal settings (Um et al., 2012; Plass et al., 2014; Le et al., 2018; Uzun and Yıldırım, 2018) were used. Future studies and meta-analyzes should take possible variations of the emotional design into account.

Another important aspect is the practicability of the emotional design, which should also be evaluated in future studies. The designs created by Bülbül and Abdullah (2021) successfully induced emotions and positively influenced educational variables are very artistic and seem extremely laborious. If such claims are made on an effective emotional design, the practical implementation (in everyday school life) seems to be difficult. Thus, the feasibility should be considered, and costs should be weighed against benefits in future research.

Future studies in the field should be based on the principle of *finding the correct balance*. Furthermore, understanding the effects and interactions of valence and arousal is still quite limited. More insights into these aspects could lead to more homogeneous results, not only about the effect on the affective state but also on educational variables. Here, it would be desirable if future emotional design research will use more (psycho)physiological measurements despite the practical challenges involved.

## Data availability statement

The raw data supporting the conclusions of this article will be made available by the authors, without undue reservation.

## Ethics statement

Ethical review and approval was not required for the study on human participants in accordance with the local legislation and institutional requirements. Written informed consent to participate in this study was provided by the participants’ legal guardian/next of kin.

## Author contributions

All authors listed have made a substantial, direct, and intellectual contribution to the work and approved it for publication.

## Funding

We acknowledge support for the Article Processing Charge from the DFG (German Research Foundation, 491454339).

## Conflict of interest

The authors declare that the research was conducted in the absence of any commercial or financial relationships that could be construed as a potential conflict of interest.

## Publisher’s note

All claims expressed in this article are solely those of the authors and do not necessarily represent those of their affiliated organizations, or those of the publisher, the editors and the reviewers. Any product that may be evaluated in this article, or claim that may be made by its manufacturer, is not guaranteed or endorsed by the publisher.
