# Supplementary material for: Emotional design pictures: Pleasant but too weak to evoke arousal and attract attention?
Source: Front Psychol. 2023 Jan 4;13:966287. doi: 10.3389/fpsyg.2022.966287 (PMC9846075; doi:10.3389/fpsyg.2022.966287)
Supplement: Supplementary file 1 [file Data_Sheet_1.PDF]

Supplementary Material

Supplementary Material S1

S1: Stimulus Material

As stimulus material, twelve central concepts covering concepts of the main topic "Lake Ecosystem" were created.

Textual elements were translated from German (original-version) to English language.

SET 1

frontiers

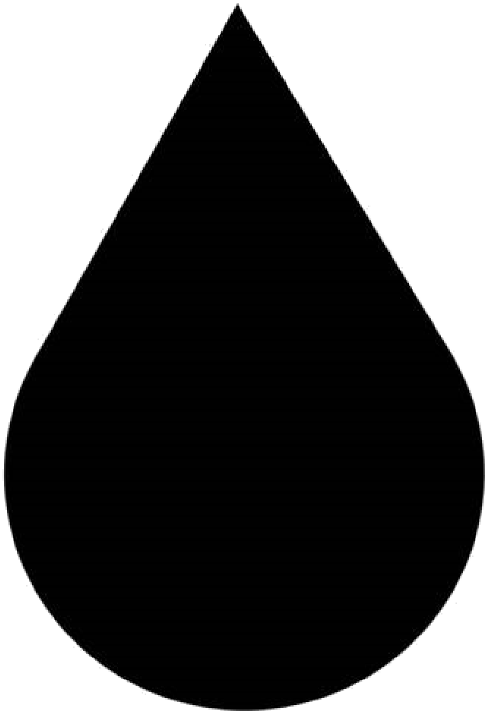

Water

frontiers

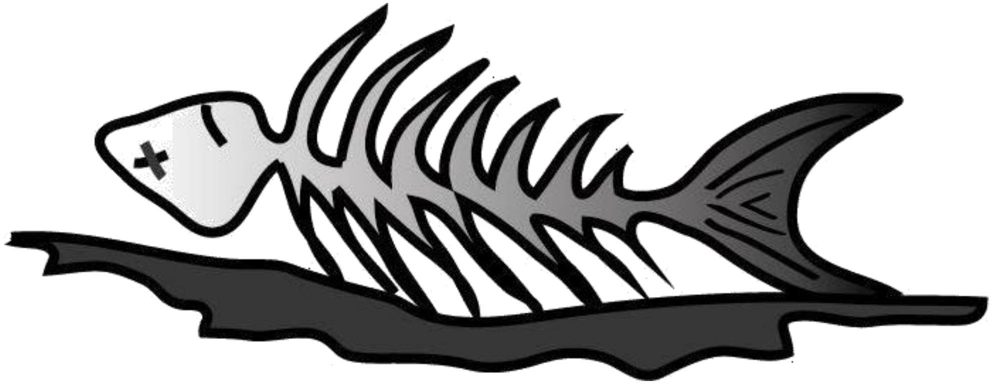

Dead consumer

frontiers

Fish

frontiers

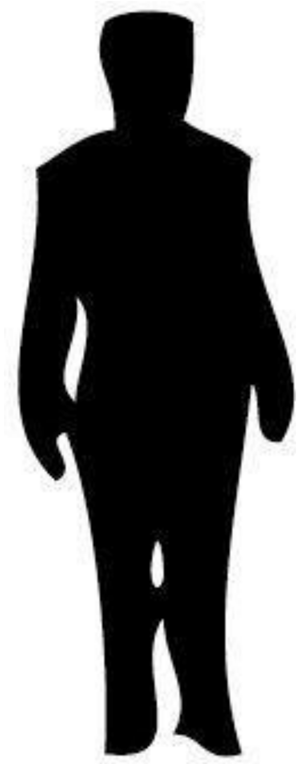

Consumer

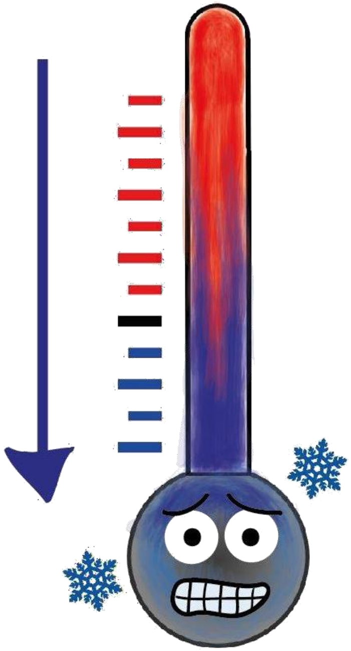

Temperature drop

Predator fish

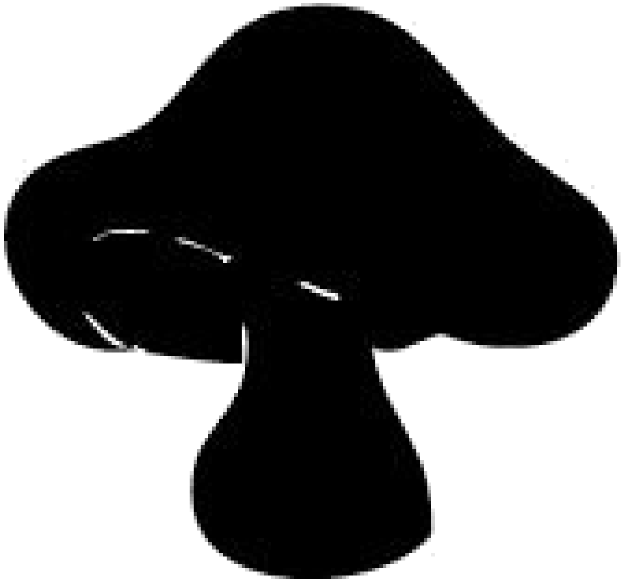

Mushroom

frontiers

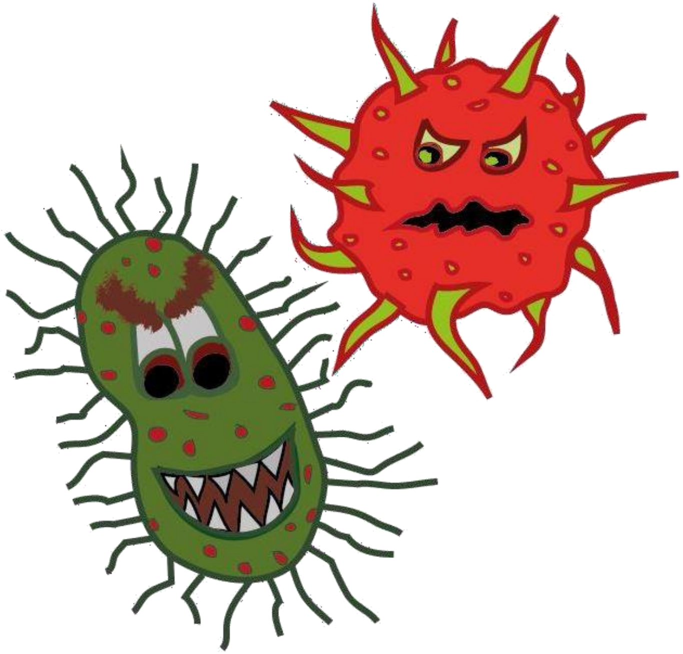

Bacteria

Plants

frontiers

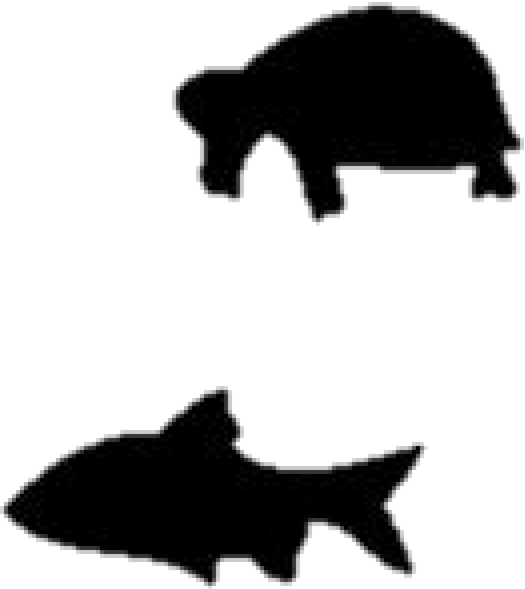

Consumers

frontiers

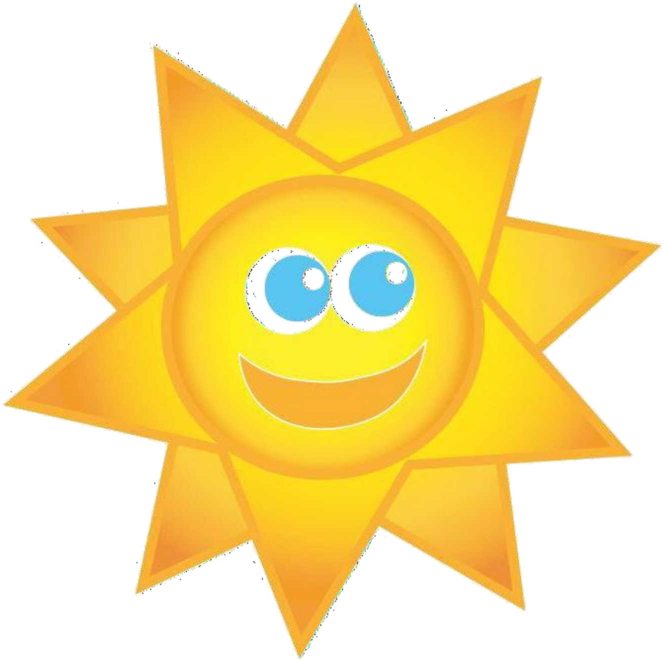

Sunlight

Temperature fluctuations

SET 2

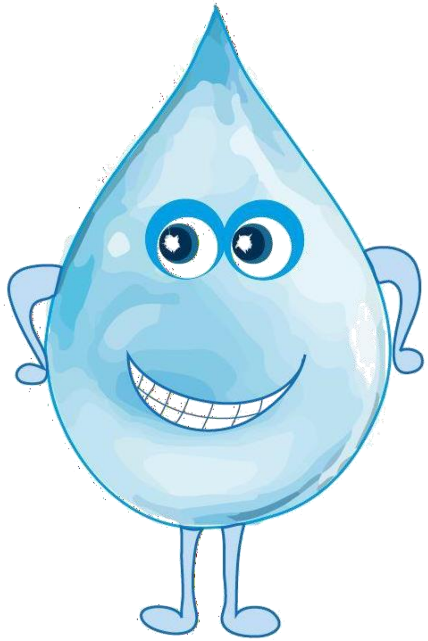

Water

Dead consumer

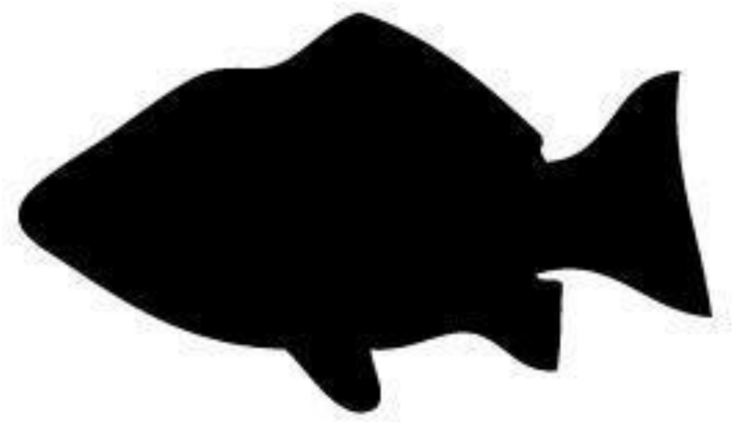

Fish

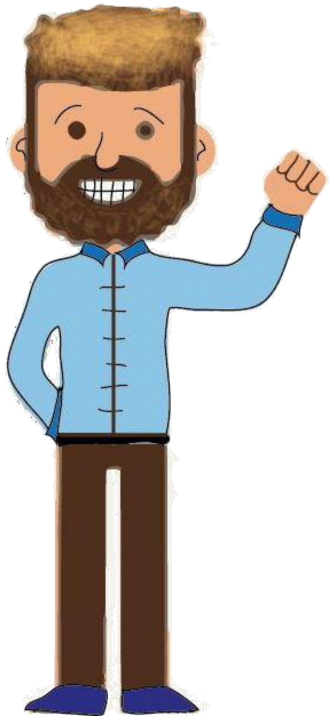

consumer

Temperature drop

frontiers

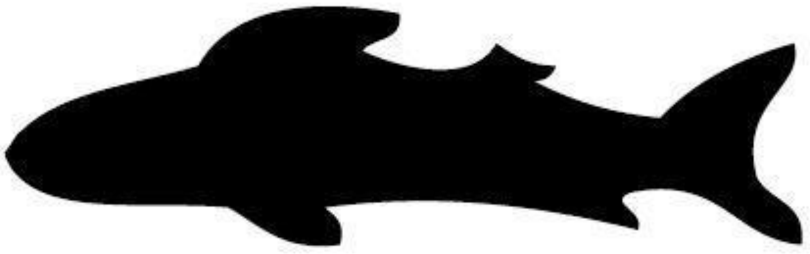

Predator fish

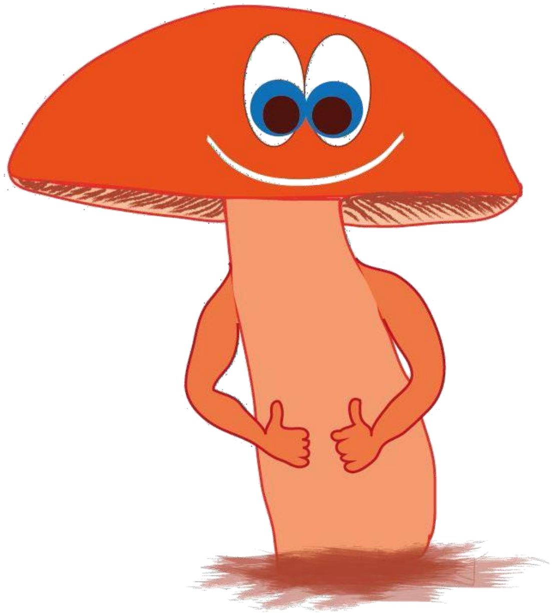

Mushroom

Bacteria

frontiers

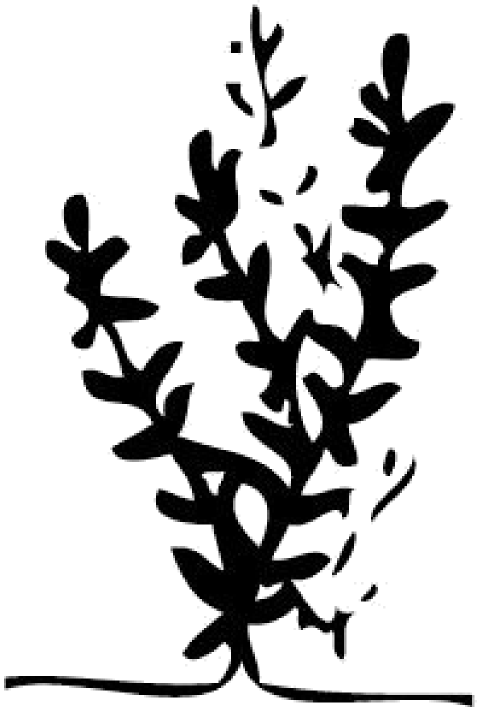

Plants

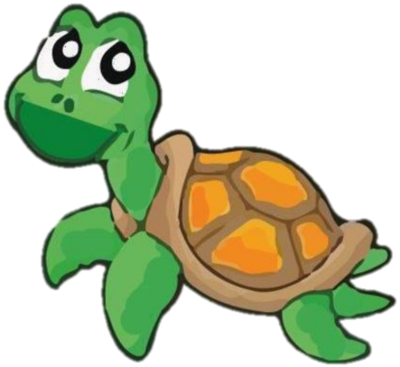

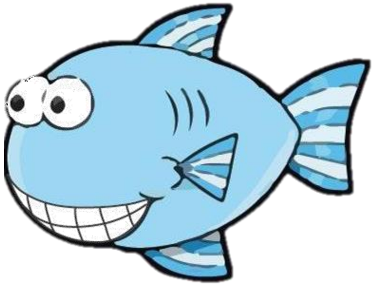

Consumers

Sunlight

frontiers

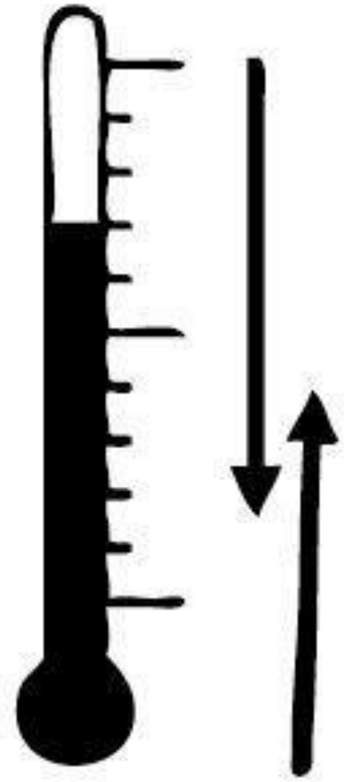

Temperature fluctuations

SET 3

Water

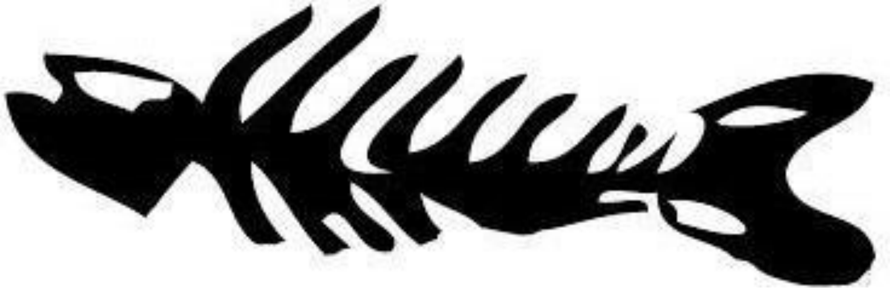

Dead consumer

frontiers

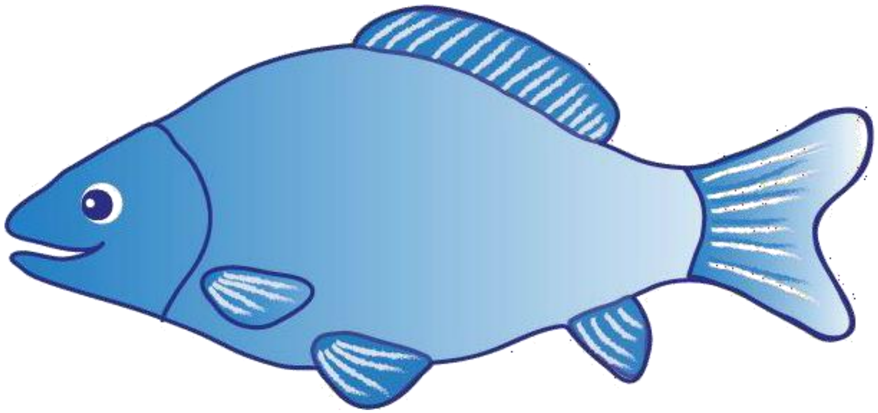

Fish

consumer

frontiers

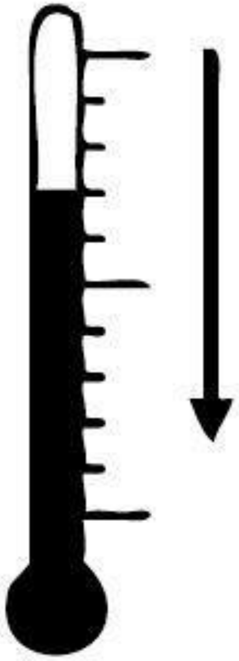

Temperature drop

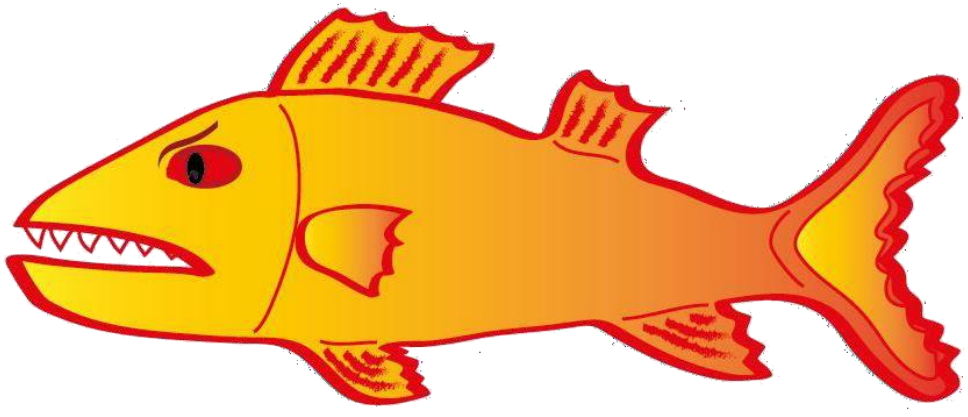

Predator fish

Mushroom

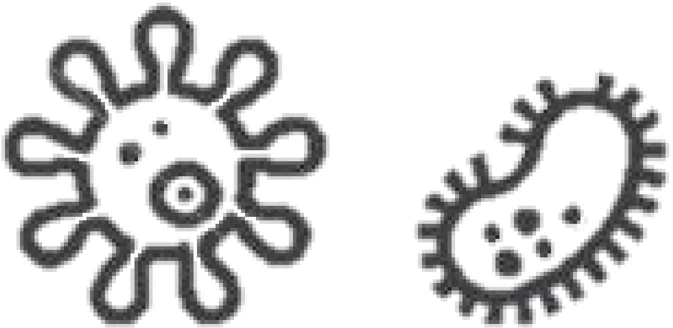

Bacteria

frontiers

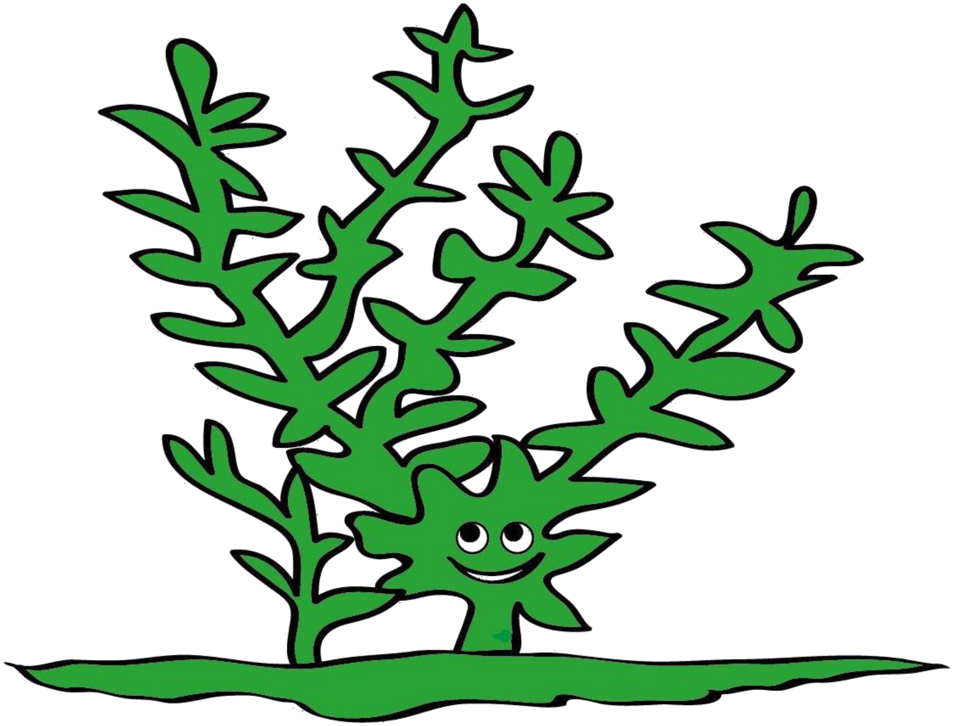

Plants

Consumers

frontiers

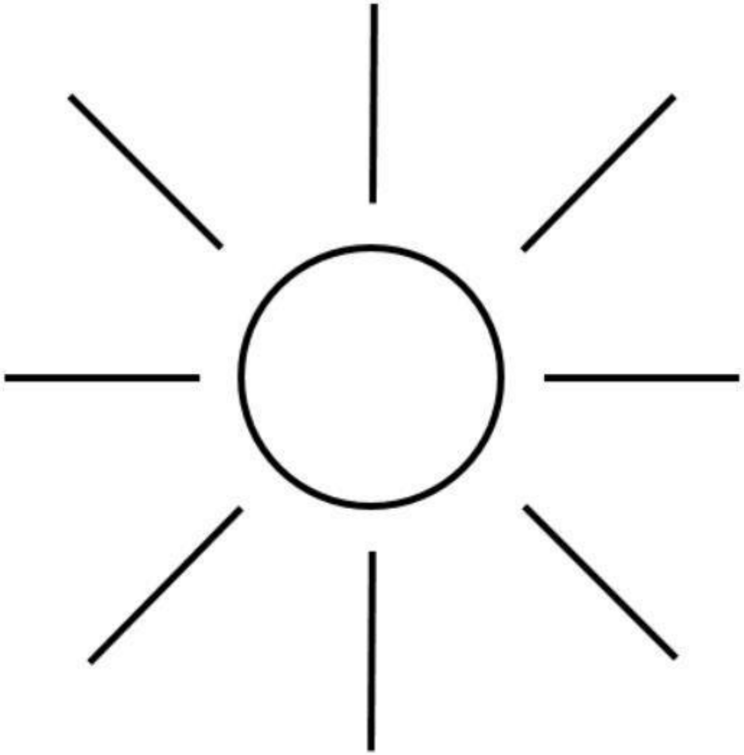

Sunlight

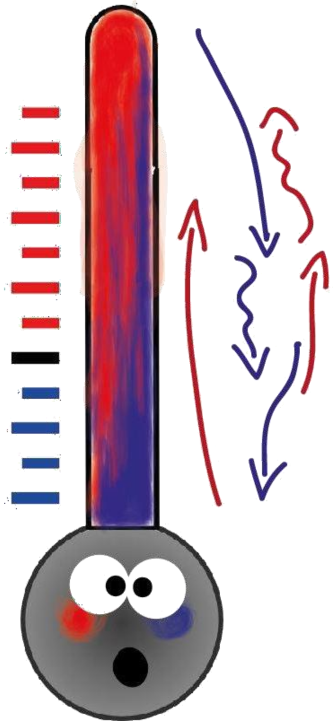

Temperature fluctuations

Supplementary Material S2

Descriptive data and post-hoc analysis of the valence ratings for the different concepts

**Concepts**		***MD***	***SD***	***p***
	Consumer	.484	.238	1.000
	Dead consumer	3.298*	.252	.000
	Fish	.429	.279	1.000
	Consumers	.129	.125	1.000
	Temperature fluctuations	1.180	.426	.989
Water	Sunlight	-.496	.255	1.000
	Bacteria	1.720*	.361	.020
	Plants	-.207	.126	1.000
	Mushrooms	.236	.104	1.000
	Predator fish	1.840*	.266	.000
	Temperature drop	1.031*	.099	.000
	Water	-.484	.238	1.000
	Dead consumer	2.813*	.259	.000
	Fish	-.056	.127	1.000
	Consumers	-.356	.190	1.000
	Temperature fluctuations	.696	.265	1.000
Consumer	Sunlight	-.980*	.168	.003
	Bacteria	1.236*	.205	.002
	Plants	-.691	.216	.420
	Mushrooms	-.249	.193	1.000
	Predator fish	1.356*	.213	.001
	Temperature drop	.547	.226	1.000
	Water	-3.298*	.252	.000
	Consumer	-2.813*	.259	.000
	Fish	-2.869*	.319	.000
	Consumers	-3.169*	.275	.000
	Temperature fluctuations	-2.118*	.393	.006
Dead consumer	Sunlight	-3.793*	.318	.000
	Bacteria	-1.578*	.252	.001
	Plants	-3.504*	.257	.000
	Mushrooms	-3.062*	.292	.000
	Predator fish	-1.458*	.241	.002
	Temperature drop	-2.267*	.219	.000
Fish	Water	-.429	.279	1.000

	Consumer	.056	.127	1.000
	Dead consumer	2.869*	.319	.000
	Consumers	-.300	.212	1.000
	Temperature fluctuations	.751	.219	.269
	Sunlight	-.924*	.170	.006
	Bacteria	1.291*	.222	.003
	Plants	-.636	.252	1.000
	Mushrooms	-.193	.238	1.000
	Predator fish	1.411*	.258	.006
	Temperature drop	.602	.260	1.000
Consumers	Water	-.129	.125	1.000
	Consumer	.356	.190	1.000
	Dead consumer	3.169*	.275	.000
	Fish	.300	.212	1.000
	Temperature fluctuations	1.051	.359	.732
	Sunlight	-.624	.202	.530
	Bacteria	1.591*	.329	.017
	Plants	-.336	.161	1.000
	Mushrooms	.107	.124	1.000
	Predator fish	1.711*	.281	.002
	Temperature drop	.902*	.137	.001
Temperature fluctuations	Water	-1.180	.426	.989
	Consumer	-.696	.265	1.000
	Dead consumer	2.118*	.393	.006
	Fish	-.751	.219	.269
	Consumers	-1.051	.359	.732
	Sunlight	-1.676*	.243	.000
	Bacteria	.540	.232	1.000
	Plants	-1.387	.425	.371
	Mushrooms	-.944	.389	1.000
	Predator fish	.660	.353	1.000
	Temperature drop	-.149	.384	1.000
Sunlight	Water	.496	.255	1.000
	Consumer	.980*	.168	.003
	Dead consumer	3.793*	.318	.000
	Fish	.924*	.170	.006
	Consumers	.624	.202	.530
	Temperature fluctuations	1.676*	.243	.000

	Bacteria	2.216*	.261	.000
	Plants	.289	.266	1.000
	Mushrooms	.731	.221	.344
	Predator fish	2.336*	.313	.000
	Temperature drop	1.527*	.250	.002
Bacteria	Water	-1.720*	.361	.020
	Consumer	-1.236*	.205	.002
	Dead consumer	1.578*	.252	.001
	Fish	-1.291*	.222	.003
	Consumers	-1.591*	.329	.017
	Temperature fluctuations	-.540	.232	1.000
	Sunlight	-2.216*	.261	.000
	Plants	-1.927*	.352	.005
	Mushrooms	-1.484*	.340	.043
	Predator fish	.120	.260	1.000
	Temperature drop	-.689	.325	1.000
Plants	Water	.207	.126	1.000
	Consumer	.691	.216	.420
	Dead consumer	3.504*	.257	.000
	Fish	.636	.252	1.000
	Consumers	.336	.161	1.000
	Temperature fluctuations	1.387	.425	.371
	Sunlight	-.289	.266	1.000
	Bacteria	1.927*	.352	.005
	Mushrooms	.442	.165	1.000
	Predator fish	2.047*	.272	.000
	Temperature drop	1.238*	.173	.000
Mushrooms	Water	-.236	.104	1.000
	Consumer	.249	.193	1.000
	Dead consumer	3.062*	.292	.000
	Fish	.193	.238	1.000
	Consumers	-.107	.124	1.000
	Temperature fluctuations	.944	.389	1.000
	Sunlight	-.731	.221	.344
	Bacteria	1.484*	.340	.043
	Plants	-.442	.165	1.000
	Predator fish	1.604*	.260	.002
	Temperature drop	.796*	.129	.002

	Water	-1.840*	.266	.000
	Consumer	-1.356*	.213	.001
	Dead consumer	1.458*	.241	.002
	Fish	-1.411*	.258	.006
	Consumers	-1.711*	.281	.002
Predator fish	Temperature fluctuations	-.660	.353	1.000
	Sunlight	-2.336*	.313	.000
	Bacteria	-.120	.260	1.000
	Plants	-2.047*	.272	.000
	Mushrooms	-1.604*	.260	.002
	Temperature drop	-.809	.247	.363
	Water	-1.031*	.099	.000
	Consumer	-.547	.226	1.000
	Dead consumer	2.267*	.219	.000
	Fish	-.602	.260	1.000
	Consumers	-.902*	.137	.001
Temperature drop	Temperature fluctuations	.149	.384	1.000
	Sunlight	-1.527*	.250	.002
	Bacteria	.689	.325	1.000
	Plants	-1.238*	.173	.000
	Mushrooms	-.796*	.129	.002
	Predator fish	.809	.247	.363

Adjustment for multiple comparisons: Bonferroni.

* The mean difference is significant at the .05 level.
